# Traditional herbal medicine combined with first-line platinum-based chemotherapy for advanced non-small-cell lung cancer: A PRISMA-compliant systematic review and meta-analysis

**DOI:** 10.1097/MD.0000000000027163

**Published:** 2021-09-17

**Authors:** Hayun Jin, Su Bin Park, Jee-Hyun Yoon, Jee Young Lee, Eun Hye Kim, Seong Woo Yoon

**Affiliations:** a College of Korean Medicine, Kyung Hee University, Seoul, Republic of Korea; b Korean Medicine Cancer Center, Kyung Hee University Hospital at Gangdong, Seoul, Republic of Korea; c Jaseng Spine and Joint Research Institute, Jaseng Medical Foundation, Seoul, Republic of Korea.

**Keywords:** meta-analysis, non-small-cell lung cancer, platinum-based chemotherapy, systematic review, traditional herbal medicine

## Abstract

**Background:**

Non-small-cell lung cancer (NSCLC) is a major health burden in many countries. This review aimed to evaluate the efficacy of traditional herbal medicine (THM) combined with first-line platinum-based chemotherapy (PBCT) for the treatment of advanced NSCLC.

**Methods:**

From inception to April 2021, relevant studies were retrieved from 9 electronic databases. Randomized controlled trials (RCTs) comparing survival outcomes of THM + PBCT treatment with PBCT treatment in patients with advanced NSCLC were reviewed. The risk of bias was evaluated using the Cochrane Risk of Bias Tool. Overall survival, 1-year survival, progression-free survival or time to progression, tumor response rate, and adverse effects were analyzed.

**Results:**

Sixteen RCTs comprising 1445 patients were included. The meta-analysis indicated that THM + PBCT treatment, compared to PBCT alone, could improve overall survival (median survival ratio = 1.24, 95% confidence intervals [CI] [1.11, 1.39], *P* < .001), progression-free survival/time to progression (median survival ratio = 1.22, 95% CI [1.09, 1.37], *P* < .001), and the 1-year survival rate (risk ratio [RR] = 1.56, 95% CI [1.31, 1.86], *P* < .001). THM + PBCT also led to a higher tumor response rate (RR = 1.39, 95% CI [1.22, 1.59], *P* < .001) and lower incidence of thrombocytopenia (RR = 0.72, 95% CI [0.56, 0.92], *P* = .009) and nausea/vomiting (RR = 0.35, 95% CI [0.21, 0.57], *P* < .001), while there was no significant effect observed on leukopenia (RR = 0.68, 95% CI [0.34, 1.36], *P* = .27).

**Conclusion:**

THM, when used in combination with PBCT, might increase survival and the tumor response rate while decreasing the side effects caused by chemotherapy in patients with advanced NSCLC. However, considering the limited methodological qualities of the included trials, more rigorous RCTs are needed.

## 1 Introduction

Lung cancer is an ongoing health concern in many parts of the world. According to the Global Cancer Observatory estimates, lung cancer accounted for the second largest number of new cases of cancer worldwide and the largest number of deaths.^[1]^ Although recent advancements have been made, especially in the early diagnosis and development of novel therapies, lung cancer remains one of the major causes of cancer-related death.

Non-small-cell lung cancer (NSCLC) is a clinical type of lung cancer that refers to histological subtypes other than small-cell lung cancer. Conventional therapies for NSCLC include resection, chemotherapy, targeted/immunotherapies, and radiation therapies. Resection offers the best chance of survival in the early stages. Targeted therapies, such as epidermal growth factor receptor-tyrosine kinase inhibitors, are also used when specific molecular markers are identified. Radiation therapy may be used alone or in combination with chemotherapy.^[2,3]^ However, in 79% of cases, cancers are already regionally or distantly metastasized at the time of their diagnosis, at a stage where curative resection is not applicable.^[4]^ In addition, targeted therapies or immunotherapies are applicable in less than half of cases.^[5,6]^ Therefore, conventional chemotherapy regimens for NSCLC rely on platinum-based agents as the first-line treatment.^[7]^

Platinum-based chemotherapy, although widely used, often causes extensive side effects, including myelosuppression, nausea and vomiting, nephrotoxicity, ototoxicity, and peripheral neuropathy.^[7]^ Although conventional therapies exist for common side effects of chemotherapy, their use is limited and may cause further side effects. The use of traditional herbal medicine (THM) in East Asia among patients with lung cancer has become relatively common. Many studies have tested the efficacy of THM in preventing and reducing the side effects of chemotherapy.^[8,9]^ Furthermore, some studies have demonstrated that some specific THM drugs may have survival benefits for NSCLC patients when used with conventional chemotherapy in general.^[10–12]^ However, the exact benefits of THM treatment when used with first-line platinum-based chemotherapy still need to be verified.

This review aims to explore the survival benefits of THM treatment alongside first-line platinum-based chemotherapy in patients with NSCLC.

## 2 Methods

### 2.1 Database and search strategy

In January 2021, we performed searches of studies in 9 electronic databases, namely PubMed, EMBASE, Cochrane Library, Chinese National Knowledge Infrastructure, Japanese databases (CiNii and J-Stage), and Korean databases (KISS, KMbase, and Oasis). Search strategies were designed to include all studies concerning lung cancer and the use of traditional herbal medicine (see Supplemental Content 1 for the search strategies used in online databases). The following keywords or Medical Subject Headings and their abbreviations or derivatives were utilized singly or in combination: lung neoplasm, lung cancer, non-small-cell lung cancer, NSCLC, traditional Chinese medicine, traditional Korean medicine, Kampo medicine, herbal medicine, randomized controlled trial, and clinical trial. The search terms were modified for the different databases using a highly sensitive search strategy developed by the Cochrane Collaboration. Language or regional restrictions were not included. Ethical approval is not required because all the research materials are published studies.

### 2.2 Inclusion and exclusion criteria

Clinical randomized controlled trials (RCTs) that met the following criteria were included:

(1) patients diagnosed with advanced NSCLC (unresectable stage IIIA and stage IIIB–IV according to the American Joint Committee on Cancer Tumor-Node-Metastasis staging) via pathological methods;
(2) treatment groups receiving first-line platinum-based chemotherapy treatment combined with oral traditional herbal medicine;
(3) control groups receiving first-line platinum-based chemotherapy;
(4) outcome measures including at least one of the following: overall survival (OS), progression-free survival (PFS), or time to progression (TTP).

Studies were excluded if they met at least one of the following conditions:

(1) studies not consistent with the main clinical question;
(2) studies that limited the enrollment criteria to patients with certain genetic mutations eligible for targeted therapies;
(3) studies in which therapeutic methods other than platinum-based chemotherapy were used;
(4) studies in which patients with a history of other chemotherapy were not excluded; and
(5) non-randomized controlled studies.

### 2.3 Data extraction and quality assessment

Two reviewers (HYJ and EHK) independently screened the articles and extracted the data by reading the full text based on the inclusion and exclusion criteria. Disagreements between the 2 reviewers were resolved by consensus or by a third reviewer (SWY). Basic data including the first author, year, enrollment criteria, number of patients enrolled in each group, baseline characteristics, interventions, and observed outcomes were retrieved.

The methodological quality of the studies was assessed by 2 reviewers. Each study was assessed based on the following criteria: random sequence generation, allocation concealment, blinding of participants and personnel, blinding of outcome assessment, incomplete outcomes, selective reporting, and other biases. Each item was assessed as high, low, or unknown risk of bias using the Cochrane risk-of-bias tool.^[13]^ Disagreements were resolved through discussion with a third reviewer.

### 2.4 Outcome measures

The primary outcome was OS. The secondary outcomes were 1-year survival rate, PFS/TTP, tumor response rate, and chemotherapy toxicity. OS was defined as the time from study medication to death due to any cause; 1-year survival rate was calculated as the number of surviving patients divided by the total at 12 months after treatment; PFS was defined as the time between treatment initiation and tumor progression or death from any cause, with censoring of patients who were lost to follow-up; TTP is defined as the time between treatment initiation and tumor progression. PFS/TTP is considered an acceptable surrogate endpoint for OS in cancer; tumor response rate was calculated as the number of patients who have a partial or complete response to therapy divided by the total number based on the Response Evaluation Criteria in Solid Tumor criteria; and chemotherapy toxicity based on the Common Terminology Criteria for Adverse Events.

### 2.5 Statistical analysis

Data analysis was performed using RevMan software (version 5.4, The Cochrane Collaboration, Oxford, UK) and R software (version 4.1.0, R Core Team, R Foundation for Statistical Computing, Vienna, Austria). The median survival ratio (MSR) was used to analyze the OS and PFS/TTP. MSR has been used in previous meta-analysis studies to analyze survival data.^[14]^ MSR was calculated by dividing the median survival time of the THM + platinum-based chemotherapy (PBCT) group by that of the PBCT group. HR and MSR were analyzed using inverse variance under a random effects model. Dichotomous outcomes were analyzed using the Mantel–Haenszel method for risk ratios (RR) under a random effects model. The effect sizes are reported as 95% confidence interval (CI) values and visualized using forest plots. *I*^2^ values were calculated to evaluate heterogeneity. If *I*^2^ was greater than 50%, it was assumed that the effects were heterogeneous. Sensitivity analyses were performed for each outcome to verify the consistency of the synthesized outcomes. Publication bias was evaluated by analyzing funnel plots. If more than 10 studies were included in the meta-analysis, Egger's test was used to assess publication bias.

## 3 Results

**Figure 1:**
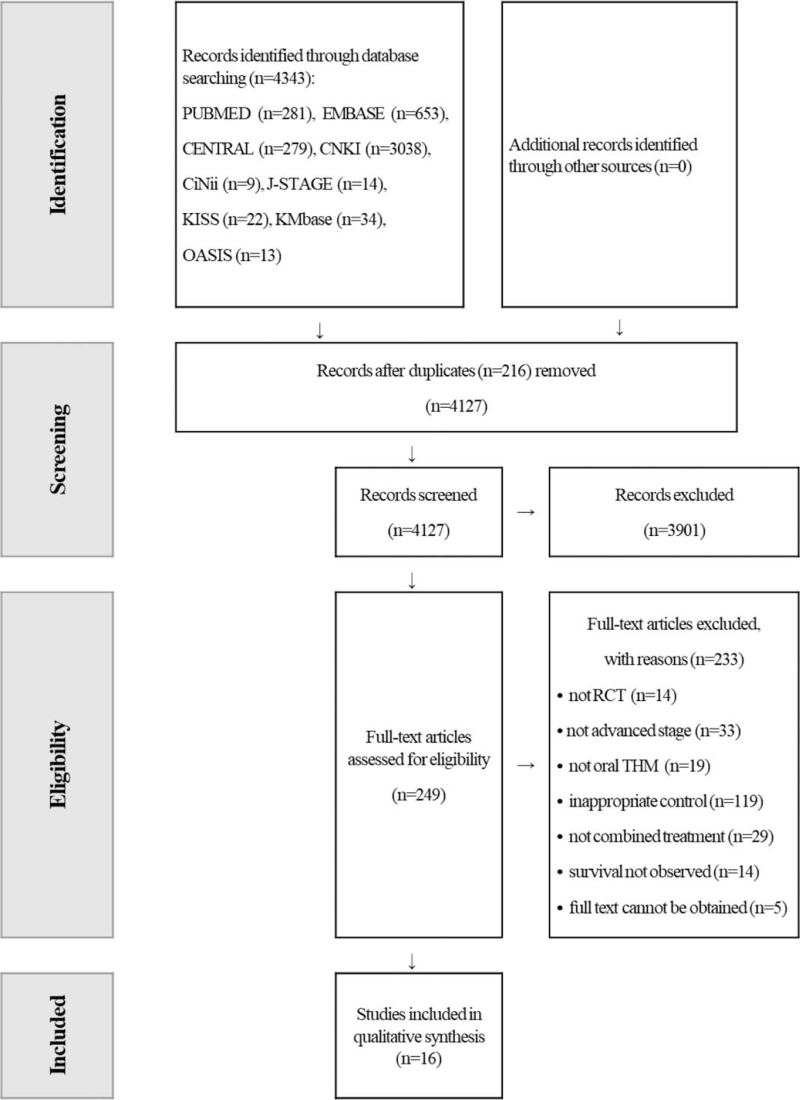
PRISMA flowchart of the study selection process. RCT = randomized controlled trial, THM = traditional herbal medicine.

In the initial search process, a total of 4343 studies were identified through online databases as potentially relevant. After removing 216 duplicates, 4127 records were screened by reading the titles and abstracts. After screening out 3901 records, 249 studies were further evaluated for eligibility by reading full texts. Next, 233 studies were excluded, with reasons. Sixteen studies met the inclusion criteria and were included in the qualitative synthesis. Figure 1 shows a flowchart of the screening and selection process.

### 3.1 Characteristics of included studies

The included studies were published between 2009 and 2021, and all were conducted in China. A total of 1445 patients were analyzed with sample sizes ranging from 59 to 200. All 16 studies recruited patients diagnosed with advanced NSCLC; 5 studies^[15–19]^ included stage IIIB–IV NSCLC patients, while 11 studies^[12,20–29]^ included patients with stage IIIA–IV NSCLC, all not subject to curative surgical resection.

All studies divided patients into PBCT + THM and PBCT groups. Fourteen studies^[15–25,27–29]^ used only oral medication as the THM treatment regimen, and 2 studies^[12,26]^ incorporated oral medications and herbal injections as THM treatment. Thirteen studies^[12,17–26,28,29]^ reported OS, 12 studies^[15–23,26,28,29]^ reported PFS/TTP, 7 studies^[12,15–17,23,25–27]^ reported 1-year survival, and 14 studies^[15–18,20–29]^ reported tumor response rates. The characteristics of each study are summarized in Table 1.

**Table 1:** Characteristics of the included trials.

Study ID	Stage	Number of patients	Age	Histologic type (T/C)	PBCT regimen	THM	Duration (d)	Outcomes
Zhang 2021	IIIB–IV	T: 40C: 40	T (70.0)C (70.0)	ADC (19/20)SCC (18/16)ASC (3/4)	Gemcitabine + cisplatin	Bupi Yifei decoction	42	PFS, RR
Han 2019	IIIA–IV	T: 46C: 46	T (61.3)C (60.7)	ADC (29/28)SCC (13/15)ASC (4/3)	Gemcitabine + cisplatin	HMPI	90	PFS, OS, RR
Song 2019	IIIA–IV	T: 39C: 39	T (57.4)C (56.0)	ADC (27/25)SCC (12/14)	Gemcitabine + cisplatin	Fuzheng Jiedu decoction	32	PFS, OS, RR
Sun 2019	IIIA–IV	T: 30C: 30	T (70.6)C (71.5)	ADC (14/13)SCC (14/15)ASC (1/2)	Paclitaxel + cisplatin	Fuzheng Runfei decoction	84	OS, PFS, RR
Lin 2017	IIIB–IV	T: 60C: 60	T (64.3)C (65.0)	ADC (32/28)SCC (20/24)ETC (8/8)	Docetaxel + cisplatin	Ovateleaf Holly Bark decoction	84	PFS, 1YS, RR
Liu 2017	IIIA–IV	T: 36C: 36	T (59.2)C (56.4)	ADC (26/25)SCC (7/10)ASC (3/1)	Docetaxel + cisplatin	Jianpi Yiqi decoction	84	OS, 1YS, TTP, RR
Wang RL 2016	IIIB–IV	T: 63C: 63	T (60.3)C (61.0)	ADC (14/15)SCC (33/29)ASC (10/11)LCC (6/8)	Gemcitabine + cisplatin	Jianpi Bufei decoction	63	OS, 1YS, TTP, RR
Wang QL 2016	IIIA–IV	T: 38C: 38	T (65.8)C (67.6)	ADC (12/10)SCC (17/19)ASC (5/7)LCC (4/2)	Vinorelbine + cisplatin	Yiqi Qingfei decoction	60	OS, RR
Li 2015	IIIA–IV	T: 43C: 43	T (51.4)C (52.5)	ADC (12/13)SCC (13/14)UDC (18/16)	Vinorelbine or gemcitabine + cisplatin	HMPI	63	OS, 1YS, RR
He 2014	IIIB–IV	T: 30C: 29	T (58.6)C (59.8)	ADC (14/10)SCC (13/18)ETC (3/1)	Gemcitabine + cisplatin	Qingjinyiqi decoction	42	OS, RR
Cui 2014	IIIA–IV	T: 40C: 40	Not described	Not described	Docetaxel + cisplatin	Liujunzi decoction	63	OS, 1YS, PFS, RR
Xi 2014	IIIA–IV	T: 34C: 33	Not described	Not described	Gemcitabine + cisplatin	Zhenqu Yiqi capsule	42–84	PFS, RR
Lin 2013	IIIA–IV	T: 49C: 49	T (63)C (64)	ADC (18/19)SCC (22/23)MUC (9/7)	Cyclophosphamide + adriamycin + cisplatin/etoposide + cisplatin/taxol + carboplatin	HMPI	42–56	OS, 1YS
Xie 2012	IIIB–IV	T: 102C: 98	T (62)C (60)	ADC (56/55)SCC (42/40)ETC (4/3)	Gemcitabine + cisplatin	Feiai prescription	42–84	OS, PFS
Xu 2011	IIIA–IV	T: 35C: 34	Not described	Not described	Docetaxel or gemcitabine + cisplatin	Huisheng oral liquid	42–126	OS, TTP, RR
Jing 2009	IIIA–IV	T: 41C: 41	Not described	ADC (15/13)SCC (24/27)ASC (2/1)	Vinorelbine + cisplatin	HMPI	56–168	OS, PFS, 1YS, RR

1YS = 1-year survival, ADC = adenocarcinoma, ASC = adenosquamous carcinoma, BAC = bronchoalveolar carcinoma, C = control group, ETC = other, HMPI = herbal medicine according to pattern identification, LCC = large cell carcinoma, MUC = magnocellular undifferentiated carcinoma, OS = overall survival, PBCT = platinum-based chemotherapy, PFS = progression-free survival, RR = tumor response rate, SCC = squamous cell carcinoma, T = treatment group, THM = traditional herbal medicine, TTP = time to progression, UDC = undifferentiated cell carcinoma.

All studies used THM prescriptions comprising multiple herbs. The compositions of THM prescriptions used in each study are listed (see Table, Supplemental Content 2, which demonstrates the composition of traditional herbal medicines used in the included studies). Astragalus (Astragali Radix: root of *Astragalus membranaceus* Bunge var. *mongholicus* Hsiao or *Astragalus membranaceus* Bunge) was the most commonly used herb and included in 11 studies.

### 3.2 Risk of bias in included studies

**Figure 2:**
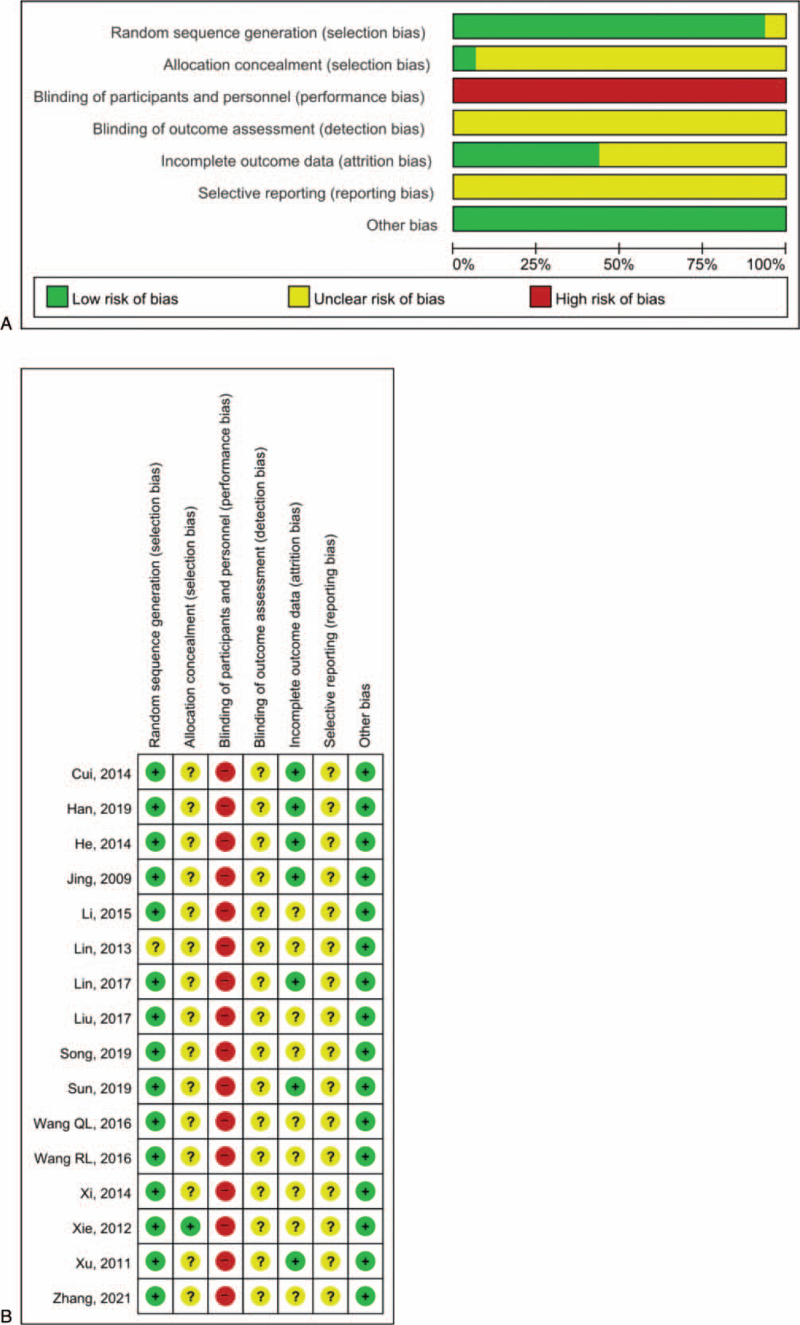
Risk of bias of the included studies – risk of bias graph (A) and summary (B).

The methodological quality of the included studies is summarized in Figure 2. One study^[12]^ did not describe the randomization method. Allocation concealment was performed in 1 study,^[19]^ and the rest did not provide sufficient information to judge selection bias. All 15 studies were open-label studies without the use of placebo and were judged as having a high risk of performance bias. No study provided information on whether the measurement of outcomes was performed by independent, blinded personnel. Seven studies^[12,15,17,19,22–25,27]^ did not report follow-up rates for each group and a low number of dropouts. All studies provided insufficient data to judge reporting bias, including pre-published protocols.

### 3.3 Overall survival

**Figure 3:**
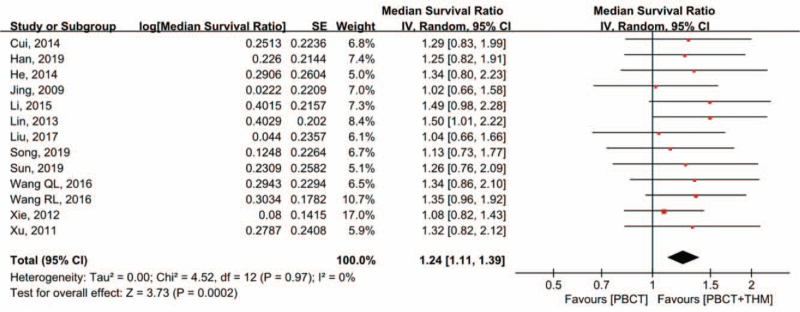
Forest plot of OS. Median survival ratio of OS compared in patients with PBCT plus THM versus PBCT alone. OS = overall survival, PBCT = platinum-based chemotherapy, THM = traditional herbal medicine.

Thirteen studies^[12,17–26,28,29]^ reported overall survival. Of these, 10 studies^[12,17–22,25,26,28]^ reported a significantly longer median survival time in the THM + PBCT group, while 4 reported no statistical difference between the 2 groups. Thirteen studies^[12,17–26,28,29]^ including 1178 patients, were eligible for quantitative synthesis of MSR. Meta-analysis of MSR showed a 24% elongation of median survival time in the combination treatment group, which was statistically significant (MSR = 1.24, 95% CI [1.11, 1.39], *P* < .001). No significant heterogeneity was observed (*I*^2^ = 0%; Fig. 3). Sensitivity analysis showed that the results were not likely to be affected by a single study.

### 3.4 Progression-free survival/time to progression

**Figure 4:**
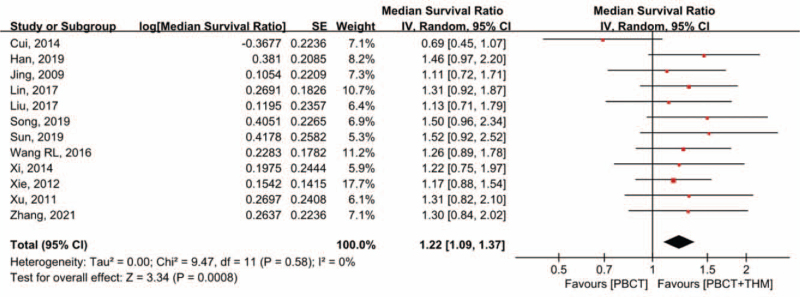
Forest plot of PFS/TTP. Median survival ratio of PFS/TTP compared in patients with PBCT plus THM versus PBCT alone. PBCT = platinum-based chemotherapy, PFS = progression-free survival, THM = traditional herbal medicine, TTP = time to progression.

Nine studies^[15,16,19–22,26,27,29]^ reported PFS, and 3 studies^[17,23,28]^ reported TTP. Ten studies^[15,17–22,26–28]^ reported significantly longer median PFS/TTP in the THM + PBCT group, while 2 reported no statistical difference between the 2 groups. Of these, 12 studies,^[15–17,19–23,26–29]^ including 1126 patients, were eligible for the quantitative synthesis of MSR. Meta-analysis of MSR showed 22% elongation of median progression-free survival, which was statistically significant (MSR = 1.22, 95% CI [1.09, 1.37], *P* < .001). No significant heterogeneity was observed (*I*^2^ = 0%) (Fig. 4). Sensitivity analysis showed that the results were not likely to be affected by a single study.

### 3.5 One-year survival rate

**Figure 5:**
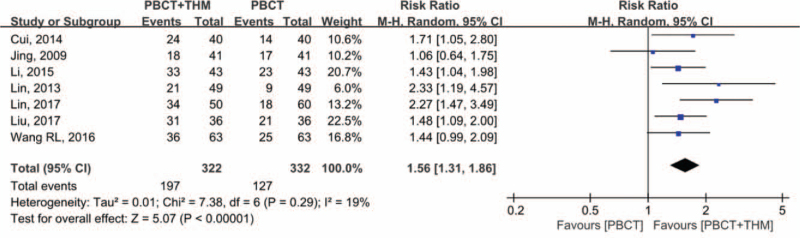
Forest plot of 1-year survival. Risk ratio of 1-year survival compared in patients with PBCT plus THM versus PBCT alone. PBCT = platinum-based chemotherapy, THM = traditional herbal medicine.

Seven studies^[12,15–17,23,25–27]^ including 654 patients reported 1-year survival rates and were included in the quantitative synthesis. Meta-analysis of risk ratios showed a statistically significant difference (RR = 1.56, 95% CI [1.31, 1.86], *P* < .001), favoring combined treatment over chemotherapy alone. There was no significant heterogeneity (*I*^2^ = 19%; Fig. 5). Sensitivity analysis showed that the results were not likely to be affected by a single study.

### 3.6 Tumor response rate

**Figure 6:**
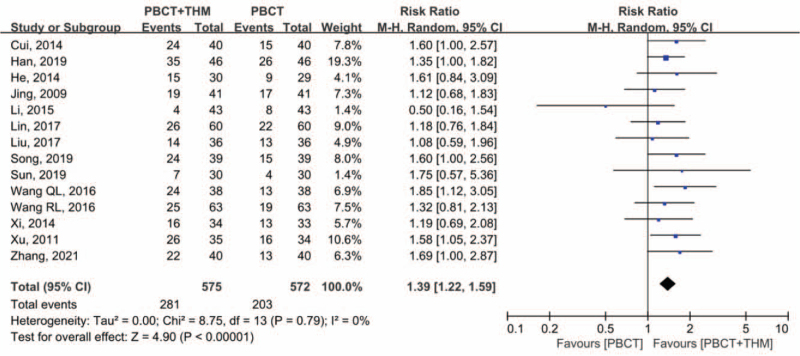
Forest plot of tumor response rate. Risk ratio of tumor response rate compared in patients with PBCT plus THM versus PBCT alone. PBCT = platinum-based chemotherapy, THM = traditional herbal medicine.

Fourteen studies^[15–25,27–29]^ reported tumor response rates. Responsive tumors were defined as the number of cases with complete response or partial response. Meta-analysis showed statistical significance (RR = 1.39, 95% CI [1.22, 1.59], *P* < .001), favoring combined treatment over chemotherapy alone. There was no significant heterogeneity (*I*^2^ = 0%; Fig. 6). Sensitivity analysis showed that the results were not likely to be affected by a single study.

### 3.7 Chemotherapy toxicity

The included studies observed a wide range of chemotherapy-related toxicities between the 2 groups. Hematological toxicities (leukopenia, thrombocytopenia, and anemia), nausea and vomiting, diarrhea, constipation, alopecia, hepatic toxicity, renal toxicity, peripheral nerve toxicity, and fatigue were observed. Of these, leukopenia, thrombocytopenia, and nausea/vomiting were eligible for meta-analysis based on the Common Terminology Criteria for Adverse Events severity grades.

**Figure 7:**
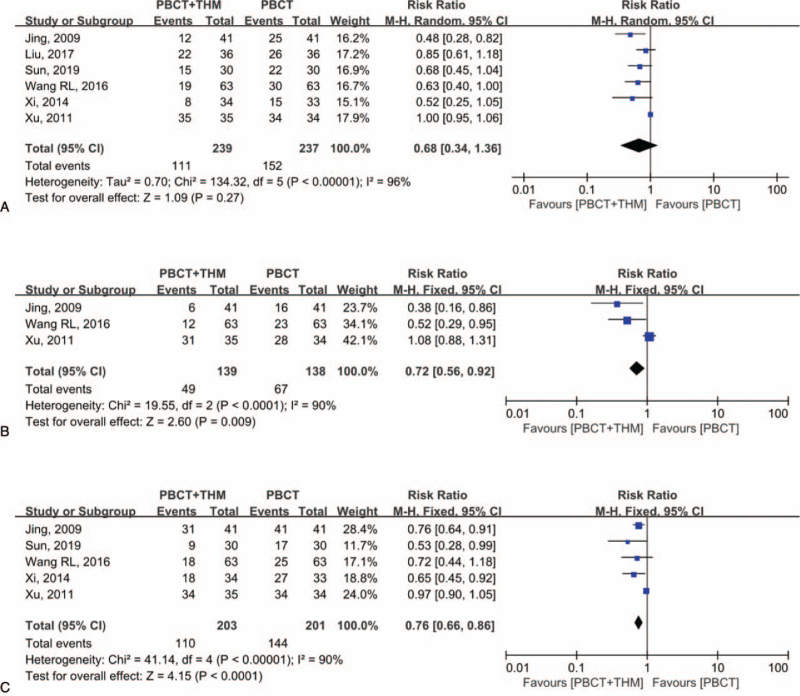
Forest plots of incidences of leukopenia (A), thrombocytopenia (B), nausea and vomiting (C). Risk ratios are compared in patients with PBCT plus THM versus PBCT alone. PBCT = platinum-based chemotherapy, THM = traditional herbal medicine.

Six studies^[17,21,23,27–29]^ including 476 patients compared the incidence of grades 3 to 4 leukopenia after treatment. Meta-analysis revealed no statistical difference between the combined treatment and chemotherapy alone groups (RR = 0.68, 95% CI [0.34, 1.36], *P* = .27). Significant heterogeneity was observed (*I*^2^ = 96%; Fig. 7A). However, sensitivity analysis showed that the results may have been affected by the results of 1 study.

Three studies^[17,28,29]^ including 277 patients compared the incidence of grades 3 to 4 thrombocytopenia after treatment. Meta-analysis suggested that combined treatment could lower the incidence of thrombocytopenia (RR = 0.72, 95% CI [0.56, 0.92], *P* = .009). Significant heterogeneity was observed (*I*^2^ = 90%; Fig. 7B). However, sensitivity analysis showed that the results might have been affected by the results of the 2 studies.

Five studies^[17,21,27–29]^ including 404 patients compared the incidence of grades 3 to 4 nausea and vomiting after treatment. Meta-analysis suggested that combined treatment could lower the incidence of nausea and vomiting (RR = 0.76, 95% CI [0.66, 0.86], *P* < .001). No significant heterogeneity was observed (*I*^2^ = 19%; Fig. 7C). Sensitivity analysis showed that the results were not likely to be affected by a single study.

### 3.8 Safety

Three studies^[14,24,29]^ reported safety outcomes. All 3 studies reported no serious adverse outcomes.

### 3.9 Publication bias

**Figure 8:**
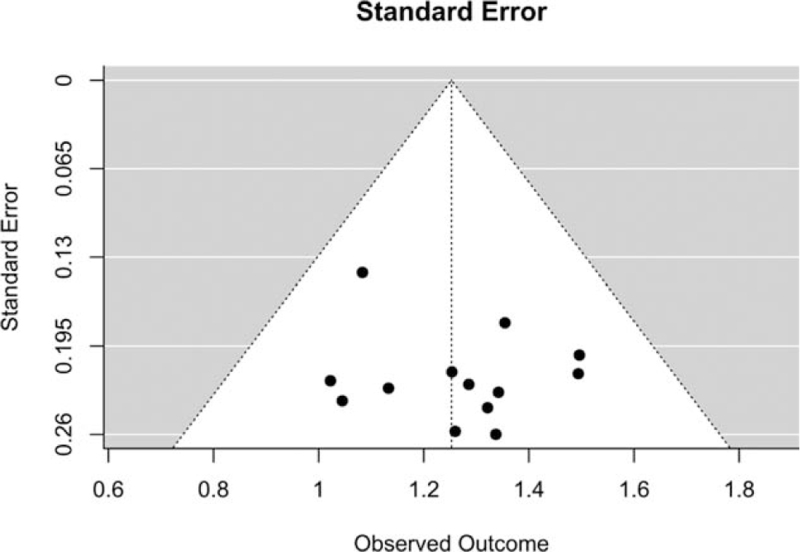
Funnel plot of the primary outcome.

A funnel plot was generated to determine the presence of publication bias. No asymmetry was observed, suggesting that publication bias was not obvious, as shown in Figure 8. Egger's test was performed for the MSR of the main outcome and indicated no significant publication bias (*Z* = .6763, *P* = .4988).

## 4 Discussion

This study analyzed 16 RCTs that included 1445 patients with NSCLC. The results from our study suggest that THM + PBCT treatment may be effective in extending OS, PFS/TTP, improving the 1-year survival rate and tumor response rate. In addition, THM was effective in reducing chemotherapy-related adverse effects, including thrombocytopenia and nausea/vomiting.

Currently, it is recommended that patients undergo molecular testing for specific driver gene mutations before commencing chemotherapy treatment. Platinum-based chemotherapy is the first treatment option for patients who do not test positive for molecular targets and/or PD-L1.^[2,3]^ In addition, molecular epidemiologic studies have shown that druggable mutations or PD-L1 positivity are found in no more than 50% of NSCLC patients.^[6,30]^ Therefore, most patients still rely on platinum-based chemotherapy as a first-line therapy. Although its efficacy and benefits are well proven, side effects of platinum-based chemotherapy, including myelosuppression, nausea and vomiting, nephrotoxicity, ototoxicity, and peripheral neuropathy, also exist, some of which may even be fatal. These side effects can negatively affect patients’ quality of life and prognosis.

Platinum-based chemotherapy refers to chemotherapy regimens in which a platinum agent (cisplatin or carboplatin) is injected in combination with a non-platinum cytotoxic agent. For patients with NSCLC included in this study, the non-platinum cytotoxic agent included taxols, gemcitabine, or vinorelbine. While clinicians may prefer some agents over others due to the differences in their toxicity profiles, previous studies have shown that there are no significant differences between regimens in terms of survival or tumor response outcomes of advanced NSCLC.^[31,32]^ Therefore, it can be assumed that the heterogeneity of chemotherapy regimens among included studies is minimal.

As an alternative and adjunctive treatment, THM is commonly used in cancer patients. THM has been shown to be effective in extending survival and improving tumor response in various types of cancer, including colorectal cancer, pancreatic cancer, nasopharyngeal cancer, hepatocellular carcinoma, and small-cell lung cancer.^[33–38]^ A meta-analysis by Wang et al^[39]^ demonstrated that astragalus-based THM + PBCT was effective in increasing overall survival and 1-, 2-, and 3-year survival rates in patients with advanced NSCLC. Wang et al^[40]^ also showed that THM + PBCT can extend the median OS and PFS in patients with advanced NSCLC. The tumor response rate is an important indicator of the effectiveness of chemotherapy.^[41]^ A meta-analysis by Li et al^[42]^ investigated the effects of THM as adjuvant therapy combined with chemotherapy and found that combination therapy led to a higher tumor response rate compared to chemotherapy alone in advanced NSCLC. Another meta-analysis by Shen et al^[43]^ showed that THM medications used for “supplementing Qi and nourishing Yin” were effective in increasing the tumor response rate in patients with advanced NSCLC. These previous studies are consistent with our meta-analysis results showing that THM might be an effective add-on option for patients with advanced NSCLC.

While the included studies utilized a wide spectrum of herbal prescriptions based on THM syndrome types, “invigorating qi” was a common principle of treatment. “Qi deficiency” is a syndrome type commonly characterized by symptoms of physical and mental fatigue, lack of appetite, and lethargy. This state of weakness not only leads to decreased quality of life but may also weaken the host's resistance to cancer.^[44]^

Astragalus (*Astragalus membranaceus* Bunge var. *mongholicus* Hsiao, *Astragalus membranaceus* Bunge) was the most commonly used herb, being included in 11 studies. Astragalus is traditionally used to treat qi deficiency. The benefits of astragalus-containing THM prescriptions have been demonstrated in several previous studies. In colorectal cancer patients, astragalus-containing THM prescriptions were effective in enhancing tumor response, improving quality of life, and reducing adverse reactions.^[45]^ In patients with NSCLC, astragalus-based THM prescriptions and astragalus injections were effective in improving survival, quality of life, performance scores, and adverse reactions.^[39,46]^

In vivo and in vitro studies have demonstrated that the bioactive ingredients of astragalus have antitumor properties by inhibiting tumor cell growth and proliferation, inducing apoptosis, inhibiting cancer migration and invasion, reducing chemotherapy resistance, and activating host immune responses, among which some were confirmed in NSCLC cells.^[47,48]^ Although THM prescriptions based on TCM theories usually comprise multiple herbs, the results from previous and current studies show that astragalus may play a key role in the benefits of THM medications.

“Removing qi stagnation and blood stasis” was another important strategy used in the included studies. In THM theory, stagnation of qi or blood is understood to cause buildups of abnormal pathological substances (“phlegm”) in the body, playing a key role in the induction of inflammatory tumorgenesis.^[44]^ Therefore, removing pathologic factors that induce stagnation and promoting the flow of qi and blood is an important principle of cancer treatment. To achieve this, the included studies used several herbs such as that promote the flow of qi and blood. Citri pericarpium (*Citrus reticulate* Blanco) and Platycodon radix (*Platycodon grandiflorum* [Jacq.] A. De Candolle), which are commonly used to promote the flow qi, were used in 6 and 3 trials, respectively. Pinelliae rhizoma (*Pinellia ternate* [Thunb.] Breit.), an herb used for removing dampness and phlegm, was included in prescriptions of 11 trials. These herbs were also experimentally found to have anticancer properties, which confirm their effectiveness in cancer treatment.^[49]^

Many experimental studies have indicated varying anticancer effects of herbal medicines against lung cancer cells. Previous reviews on experimental studies have identified at least 10 to 15 THM herbs or compounds as having substantial amounts of cytotoxicity against lung cancer cells.^[50,51]^ In this context, it can be assumed that the survival benefits of THM medication may in part be attributed to this antitumor activity. Also, a study by Guo et al reviewed molecular actions of THM ingredients or prescriptions and found that some of the substances may alter cancer cell metabolism in ways that increase the cells’ susceptibility to chemotherapeutic drugs.^[52]^ These chemosensitizing properties may partially explain the benefits of THM medications shown in this study. Therefore, more research is needed to confirm the roles and effects of herbal components in human patients with NSCLC.

Traditionally, clinical practice of THM has relied mostly on theories from literature or clinicians’ experience. However, recent advancements in THM research have made it possible to build evidence for better clinical decisions. Recent clinical trials are now adopting double-blind placebo control RCT methods for higher-quality evidence.^[53]^ Evidence-based guidelines are also being published. In China, the Clinical Practice Guidelines of Chinese Medicine in Oncology is implemented in clinical and research settings. The guideline suggests specific evidence-based interventions including THM for patients according to the cancer types, stages, main symptoms, and patient conditions. In this context, our study will provide evidence base for THM treatment in advanced NSCLC patients.

Our study has some limitations, mainly related to the lack of high-quality trials. First, many of the included studies contained possibilities of bias in the study design. None of the studies were double-blind RCTs. Although THM research has difficulties in preparing adequate placebo because of the smell, color, and taste of traditional preparations, more studies are needed to investigate the possibility and size of placebo effects. In order to minimize methodological bias, the authors used objective outcomes including survival time and tumor response rates, which are less likely to be influenced by placebo effects.^[54]^ Second, the included studies were still heterogeneous to some extent, especially with respect to differences in treatment methods. THM, by its nature, emphasizes a patient-centered approach and is currently practiced in such ways. Therefore, the methods of treatment and specific herbs used in each study have a wide spectrum. Third, all included trials took place in China, which makes it difficult to generalize the results of this study to global populations. Therefore, more rigorous RCTs with stricter study designs are needed to confirm the exact benefits of THM treatment.

## Author contributions

**Conceptualization:** Hayun Jin, Jee Young Lee, Seong Woo Yoon.

**Data curation:** Hayun Jin, Su Bin Park, Jee-Hyun Yoon, Eun Hye Kim.

**Formal analysis:** Hayun Jin, Su Bin Park, Jee-Hyun Yoon, Eun Hye Kim.

**Funding acquisition:** Seong Woo Yoon.

**Investigation:** Hayun Jin, Su Bin Park, Jee-Hyun Yoon, Eun Hye Kim.

**Methodology:** Hayun Jin, Jee Young Lee, Eun Hye Kim, Seong Woo Yoon.

**Project administration:** Seong Woo Yoon.

**Resources:** Jee Young Lee.

**Software:** Jee Young Lee, Eun Hye Kim.

**Supervision:** Seong Woo Yoon.

**Validation:** Su Bin Park, Jee-Hyun Yoon, Jee Young Lee, Eun Hye Kim.

**Visualization:** Hayun Jin.

**Writing – original draft:** Hayun Jin.

**Writing – review & editing:** Jee Young Lee, Eun Hye Kim, Seong Woo Yoon.

## Supplementary Material

Supplemental Digital Content

## Supplementary Material

Supplemental Digital Content
