# Supplementary material for: Traditional herbal medicine combined with first-line platinum-based chemotherapy for advanced non-small-cell lung cancer: A PRISMA-compliant systematic review and meta-analysis
Source: Medicine (Baltimore). 2021 Sep 17;100(37):e27163. doi: 10.1097/MD.0000000000027163 (PMC8448030; doi:10.1097/MD.0000000000027163)
Supplement: Supplemental Digital Content [file medi-100-e27163-s001.docx]

**Supplemental Content 1. Search strategies used in online databases**

**1. PubMed**

**#1** Lung Neoplasms[MeSH Terms]

**#2** Bronchopulmonary carcino*[Title/Abstract] OR Cancer of Lung*[Title/Abstract] OR Cancer of the Lung*[Title/Abstract] OR Lung adenocarcimoma*[Title/Abstract] OR Lung Cancer*[Title/Abstract] OR Lung carcinoma*[Title/Abstract] OR Lung malignan*[Title/Abstract] OR Lung Neoplasm*[Title/Abstract] OR Lung Tumo*[Title/Abstract] OR Pulmonary adenocarcinoma*[Title/Abstract] OR Pulmonary Cancer*[Title/Abstract] OR pulmonary carcino*[Title/Abstract] OR pulmonary malignan*[Title/Abstract] OR Pulmonary Neoplasm*[Title/Abstract] OR Pulmonary tumo*[Title/Abstract]

**#3** Carcinoma, Non-Small-Cell Lung[MeSH Terms] OR Nonsmall Cell Lung Cancer*[Title/Abstract] OR Non Small Cell Lung Cancer*[Title/Abstract] OR Nonsmall Cell Lung Carcinoma*[Title/Abstract] OR Non Small Cell Lung Carcinoma*[Title/Abstract] OR NSCLC [Title/Abstract]

**#4** #1 OR #2 OR #3

**#5** "Drugs, Chinese Herbal"[MeSH] OR "Plants, Medicinal"[MeSH] OR "Herbal Medicine"[MeSH] OR "Medicine, Kampo"[MeSH] OR "Medicine, Korean Traditional"[MeSH] OR "Medicine, Chinese Traditional"[MeSH]

**#6** "traditional Korean medicine"[tiab] OR "traditional Chinese medicine"[tiab] OR "traditional oriental medicine"[tiab] OR "Kampo medicine"[tiab] OR herb*[tiab] OR decoction*[tiab] OR botanic*[tiab]

**#7** #5 OR #6

**#8** #4 AND #7

**#9** (randomized controlled trial[pt] OR controlled clinical trial[pt] OR randomized[tiab] OR placebo[tiab] OR clinical trials as topic[mesh: noexp] OR randomly[tiab] OR trial[ti]) NOT (animals[mh] NOT (humans[mh] AND animals[mh]))

**#10** #8 AND #9

**2. EMBASE**

**#1** 'lung cancer'/exp

**#2** 'lung non small cell cancer'/exp

**#3** 'non small cell':ti,ab OR 'nsclc':ti,ab

**#4** #1 OR #2 OR #3

**#5** 'medicine, chinese traditional'/exp

**#6** 'drugs, chinese herbal'/exp OR 'plant extracts'/exp

**#7** 'herbal medicine'/exp OR 'plants, medicinal'/exp

**#8** #5 OR #6 OR #7

**#9** #4 AND #8

**#10** 'crossover procedure'/exp OR 'double-blind procedure'/exp OR 'randomized controlled trial'/exp OR 'single-blind procedure'/exp OR random* OR factorial* OR crossover* OR (cross NEXT/1 over*) OR placebo* OR (doubl* NEAR/1 blind*) OR (singl* NEAR/1 blind*) OR assign* OR allocat* OR volunteer*

**#11** animal NOT human

**#12** #10 NOT #11

#13 #9 AND #12

**3. Cochrane Library**

**#1** MeSH descriptor: [Lung Neoplasms] explode all trees

**#2** Lung cancer

**#3** Non small cell lung cancer

**#4** NSCLC

**#5** Pulmonary neoplasm

**#6** OR / 1-5

**#7** MeSH descriptor: [Medicine, Chinese Traditional] explode all trees

**#8** Traditional Chinese medicine

**#9** Traditional Korean medicine

**#10** Kampo medicine

**#11** Traditional Oriental medicine

**#12** Herbal medicine

**#13** OR / 7-12

**#14** #6 AND #13

**4. China National Knowledge Infrastructure**

**#1** 肺癌

**#2** 非小细胞肺癌

**#3** 肺肿瘤

**#4** lung cancer

**#5** lung neoplasm

**#6** non small cell lung cancer

**#7** NSCLC

**#8** OR / 1-7

**#9** 中药

**#10** 中医

**#11** 汤

**#12** 饮

**#13** 散

**#14** 汤剂

**#15** 丸

**#16** 中成药

**#17** 方剂

**#18** 中西医结合

**#19** 颗粒

**#20** 胶囊

**#21** 口服液

**#22** Capsule

**#23** Powder

**#24** Herbal medicine

**#25** Traditional Chinese Medicine

**#26** TCM

**#27** Traditional medicine

**#28** Decoction

**#29** Chinese medicine

**#30** OR / 9-29

**#31** 化疗

**#32** 药物治疗

**#33** 卡铂

**#34** 顺铂

**#35** 铂

**#36** chemotherapy

**#37** cisplatin

**#38** carboplatin

**#39** platininum

**#40** platin

**#41** OR / 31-40

**#42** 随机

**#43** 对照

**#44** Randomized trials

**#45** clinical

**#46** trial

**#47** research

**#48** random

**#49** randomized

**#50** OR / 42-49

**#51** #8 AND #30 AND #41 AND #50

**5. CiNii**

**#1** lung cancer OR non-small-cell lung cancer OR NSCLC OR 肺がん

**#2** Kampo OR 漢方OR herb OR extract OR plant OR decoction OR traditional medicine

**#3** randomized controlled trial OR trial OR 対照比較試験 OR 試み

**#4** #1 AND #2 AND #3

**6. J-Stage**

**#1** lung cancer OR non-small-cell lung cancer OR NSCLC OR 肺がん

**#2** Kampo OR 漢方OR herb OR extract OR plant OR decoction OR traditional medicine

**#3** randomized controlled trial OR trial OR 対照比較試験 OR 試み

**#4** #1 AND #2 AND #3

**7. KMbase**

**#1** ([ALL=lung cancer] OR [ALL=non small cell lung cancer] OR [ALL=NSCLC] OR [ALL=폐암])

**#2** ([ALL=traditional medicine] OR [ALL =korean medicine]) OR [ALL =herb] OR [ALL=한약])

**#3** #1 AND #2

**8. KISS**

**#1** lung cancer OR non small cell lung cancer OR NSCLC OR 폐암

**#2** Korean medicine OR traditional medicine OR herb OR한약

**#3** #1 AND #2

**9. OASIS**

**#1** lung cancer

**#2** non small cell lung cancer

**#3** NSCLC

**#4** 폐암

**#5** OR / 1-4
