# Supplementary material for: Traditional herbal medicine combined with first-line platinum-based chemotherapy for advanced non-small-cell lung cancer: A PRISMA-compliant systematic review and meta-analysis
Source: Medicine (Baltimore). 2021 Sep 17;100(37):e27163. doi: 10.1097/MD.0000000000027163 (PMC8448030; doi:10.1097/MD.0000000000027163)
Supplement: Supplemental Digital Content [file medi-100-e27163-s002.docx]

Supplemental Content 2.

Table S2. The composition of traditional herbal medicine in the included studies.

| **Study ID** | **Name of prescription** | **Composition** |
| --- | --- | --- |
| **Zhang**  **2021** | Bupi Yifei decoction | Codonopsis Radix, Poria, Crataegi Fructus, Massa Medicata Fermentata, Hordei Fructus Germinatus, Atractylodis Macrocephalae Rhizoma, Eupatorii Herba, Platycodonis Radix, Agastachis Herba, Aurantii Fructus, Astragali Radix, Citri Reticulatae Pericarpium, Pinelliae Rhizoma, Glycyrrhizae Radix et Rhizoma, Perillae Caulis |
| **Han**  **2019** | Herbal medicine according to pattern identification | **Yin deficiency and internal heat syndrome**: Hordei Fructus Germinatus, Setariae Fructus Germinatus, Glehniae Radix, Galli Gigerii Endothelium Corneum, Lablab Semen Album, Ophiopogonis Radix, Rehmanniae Radix, Fritillariae Thunbergii Bulbus, Mori Folium, Polygonati Odorati Rhizoma, Glycyrrhizae Radix et Rhizoma, Platycodonis Radix  **Spleen lung qi deficiency**: Hordei Fructus Germinatus, Setariae Fructus Germinatus, Galli Gigerii Endothelium Corneum, Codonopsis Radix, Pinelliae Rhizoma, Citri Reticulatae Pericarpium, Poria, Atractylodis Macrocephalae Rhizoma  **Qi and yin deficiency**: Hordei Fructus Germinatus, Setariae Fructus Germinatus, Glehniae Radix, Lablab Semen Album, Galli Gigerii Endothelium Corneum, Ophiopogonis Radix, Fritillariae Thunbergii Bulbus, Mori Folium, Polygonati Odorati Rhizoma, Schisandrae Chinensis Fructus, Glycyrrhizae Radix et Rhizoma  **Qi stagnation and blood stasis**: Hordei Fructus Germinatus, Setariae Fructus Germinatus, Galli Gigerii Endothelium Corneum, Trichosanthis Radix, Aurantii Fructus, Bupleuri Radix, wine-treated Rhei Radix et Rhizoma, Angelicae Sinensis Radix, Persicae Semen |
| **Song**  **2019** | Fuzheng Jiedu decoction | Astragali Radix, Oldenlandia diffusa Herba, Atractylodis Macrocephalae Rhizoma, Inulae Flos, Akebiae Fructus, Solani Nigri Herba, Bambusae Caulis in Taenias, Solanum lyratum, Ginseng Radix et Rhizoma, Ganoderma sinense, Pinelliae Rhizoma Praeparatum cum Zingibere et Alumine, Citri Reticulatae Pericarpium, Citri Sarcodactylis Fructus, Amomi Fructus |
| **Sun**  **2019** | Fuzheng Runfei decoction | Astragali Radix, Codonopsis Radix, Epimedii Folium, Corni Fructus, Ligustri Lucidi Fructus, Ophiopogonis Radix, Adenophorae Radix, Dendrobii Caulis, Trichosanthis Radix, Armeniacae Semen Amarum, Mori Cortex, Peucedani Radix, Houttuyniae Herba, Pinelliae Rhizoma, Fritillariae Thunbergii Bulbus, Trichosanthis Fructus, Bombyx Batryticatus, Vespae Nidus, Glycyrrhizae Radix et Rhizoma Praeparata cum Melle |
| **Lin**  **2017** | Ovateleaf Holly Bark decoction | Ilicis Rotundae Cortex, Selaginella uncinate, Coicis Semen, Adenophorae Radix, Forsythiae Fructus, Galli Gigerii Endothelium Corneum, Gecko, Fritillariae Thunbergii Bulbus, Asteris Radix et Rhizoma, Persicae Semen, Armeniacae Semen Amarum, Crataegi Fructus, Glycyrrhizae Radix et Rhizoma |
| **Liu**  **2017** | Jianpi Yiqi decoction | Astragali Radix, Codonopsis Radix, Poria, Atractylodis Macrocephalae Rhizoma, Coicis Semen, Amomi Fructus, Pinelliae Rhizoma Praeparatum, Sparganii Rhizoma, Curcumae Rhizoma |
| **Wang RL**  **2016** | Jianpi Bufei decoction | Astragali Radix, Codonopsis Radix, Citri Reticulatae Pericarpium, Pinelliae Rhizoma, Poria, Atractylodis Macrocephalae Rhizoma, Ophiopogonis Radix, Adenophorae Radix, Glycyrrhizae Radix et Rhizoma |
| **Wang QL**  **2016** | Yiqi Qingfei decoction | Pseudostellariae Radix, Astragali Radix, Schisandrae Chinensis Fructus, Citri Reticulatae Pericarpium, Eriobotryae Folium, Curcumae Radix, Mori Cortex, Pinelliae Rhizoma, Armeniacae Semen Amarum, Ophiopogonis Radix, Hedyotidis Diffusae Herba, Trichosanthis Fructus |
| **Li**  **2015** | Hechan tablet,  Shenyi capsule,  Herbal medicine according to pattern identification | **Hechan Tablet**: Hairyvein Agrimonia Herd, Corium bufonis, Catclaw Buttercup Root, Tthunberg Fritillary Bulb, Raw Pinellia, Heartleaf Houttuynia Herb, Cochinchinese Asparagus Root, Ginseng Root, Pepperweed Seed.  **Shenyi Capsule**: Ginsenoside Rg3  **Stagnation of lung, phlegm and dampness**: Prunus mandshurica Semen, Trichosanthis Fructus, Fritillariae Thunbergii Bulbus, Arisaematis Rhizoma, Pinelliae Rhizoma, Eumeces Chinensis, Coicis Semen, Prunellae Spica, Notoginseng Radix et Rhizoma  **Qi deficiency and phlegm dampness**: Trichosanthis Fructus, Fritillariae Thunbergii Bulbus, Polyporus, Poria, Codonopsis Radix, Arisaematis Rhizoma, Pinelliae Rhizoma, Eumeces Chinensis, Coicis Semen, Platycodonis Radix, Atractylodis Macrocephalae Rhizoma  **Yin deficiency and phlegm heat**: Prunellae Spica, Platycodonis Radix, Fritillariae Thunbergii Bulbus, Adenophorae Radix, Ophiopogonis Radix, Eumeces Chinensis, Coicis Semen, Agrimoniae Herba, Polyporus, Rehmanniae Radix  **Qi and yin deficiency**: Lilii Bulbus, Adenophorae Radix, Panacis Quinquefolii Radix, Codonopsis Radix, Ophiopogonis Radix, Schisandrae Chinensis Fructus, Eumeces Chinensis, Agrimoniae Herba, Platycodonis Radix, Fritillariae Thunbergii Bulbus, Polyporus |
| **He**  **2014** | Qingjinyiqi decoction | Astragali Radix, Rehmanniae Radix, Anemarrhenae Rhizoma, Glycyrrhizae Radix et Rhizoma, Scrophulariae Radix, Adenophorae Radix, Fritillariae Cirrhosae Bulbus, Arctii Fructus |
| **Cui**  **2014** | Liujunzi decoction,  Sashen maidong decoction  Yadanzi youru injection | **Liujinzi decoction**: Ginseng Radix et Rhizoma, Atractylodis Macrocephalae Rhizoma, Poria, Pinelliae Rhizoma, Citri Reticulatae Pericarpium, Glycyrrhizae Radix et Rhizoma Praeparata cum Melle, Massa Medicata Fermentata, Crataegi Fructus, Hordei Fructus Germinatus  **Sashen maidong decoction**: Adenophorae Radix, Polygonati Odorati Rhizoma, Glycyrrhizae Radix et Rhizoma, Mori Folium, Ophiopogonis Radix, Lablab Semen Album, Trichosanthis Radix  **Yadanzi youru injection**: Refined Brucea Javanica oil, refined lecithin |
| **Xi**  **2014** | Zhenqu Yiqi capsule | Astragali Radix, Codonopsis Radix, Crataegi Fructus, Atractylodis Macrocephalae Rhizoma, Poria, Citri Reticulatae Pericarpium, Ligustri Lucidi Fructus, Psoraleae Fructus, Spatholobi Caulis, Cuscutae Semen, Hordei Fructus Germinatus, Massa Medicata Fermentata, Crataegi Fructus |
| **Lin**  **2013** | Herbal medicine according to pattern identification | **Qi-deficiency and phlegm-dampness**: Astragali Radix, Citri Reticulatae Pericarpium, Pinelliae Rhizoma, Codonopsis Radix, Poria, Trichosanthis Fructus et Semen, Atractylodis Macrocephalae Rhizoma, Glycyrrhizae Radix et Rhizoma Praeparata  **Yin-deficiency and interior heat**: Ophiopogonis Radix, Adenophorae Radix, Lonicerae Japonicae Flos, Chrysanthemi Indici Flos, Taraxaci Herba, Scutellariae Barbatae Herba, Hedyotidis Diffusae Herba, Trichosanthis Radix, Glycyrrhizae Radix et Rhizoma  **Deficiencies of both Qi and Yin**: Pseudostellariae Radix, Ophiopogonis Radix, Astragali Radix, |
| **Xie**  **2012** | Feiai prescription | Houttuyniae Herba, Scutellariae Radix, Fritillariae Thunbergii Bulbus, Phragmitis Rhizoma, Asparagi Radix, Adenophorae Radix, Astragali Radix, Poria, Coicis Semen, Oldenlandia Diffusa Herba, Scutellariae Barbatae Herba, Actinidae Chinesis Radix, Ganoderma, Wenyujin Rhizoma Concisum |
| **Xu**  **2011** | Huisheng oral liquid | Ginseng Radix et Rhizoma, Codonopsis Radix, Chuanxiong Rhizoma, Carthami Flos, Leonuri Herba, Sparganii Rhizoma, Curcumae Rhizoma, Cyperi Rhizoma, Rhei Radix et Rhizoma, Trogopterori Faeces, Trionycis Carapax, Hirudo, Caryophylli Flos |
| **Jing**  **2009** | Herbal medicine according to pattern identification | Astragali Radix, Codonopsis Radix, Atractylodis Macrocephalae Rhizoma, Poria, Polygonati Rhizoma, Ligustri Lucidi Fructus, Spatholobi Caulis, Asini Corii Colla, Coicis Semen, Lablab Semen, Citri Reticulatae Pericarpium, Pinelliae Rhizoma, Zingiberis Rhizoma Recens |
